# Generalized Chirp Scaling Combined with Baseband Azimuth Scaling Algorithm for Large Bandwidth Sliding Spotlight SAR Imaging

**DOI:** 10.3390/s17061237

**Published:** 2017-05-29

**Authors:** Tianzhu Yi, Zhihua He, Feng He, Zhen Dong, Manqing Wu

**Affiliations:** 1 School of Electronic Science and Engineering, National University of Defense Technology, Sanyi Avenue, Changsha 410073, China; skynismile@163.com (Z.H.); hefeng@nudt.edu.cn (F.H.); dongzhen@nudt.edu.cn (Z.D.); 2 China Electronics Technology Group Corporation (CETC), China Academy of Electronics and Information Technology, Beijing 100846, China; wumanqing@ustc.edu.cn

**Keywords:** generalized chirp scaling, baseband azimuth scaling, large bandwidth, sliding spotlight

## Abstract

This paper presents an efficient and precise imaging algorithm for the large bandwidth sliding spotlight synthetic aperture radar (SAR). The existing sub-aperture processing method based on the baseband azimuth scaling (BAS) algorithm cannot cope with the high order phase coupling along the range and azimuth dimensions. This coupling problem causes defocusing along the range and azimuth dimensions. This paper proposes a generalized chirp scaling (GCS)-BAS processing algorithm, which is based on the GCS algorithm. It successfully mitigates the deep focus along the range dimension of a sub-aperture of the large bandwidth sliding spotlight SAR, as well as high order phase coupling along the range and azimuth dimensions. Additionally, the azimuth focusing can be achieved by this azimuth scaling method. Simulation results demonstrate the ability of the GCS-BAS algorithm to process the large bandwidth sliding spotlight SAR data. It is proven that great improvements of the focus depth and imaging accuracy are obtained via the GCS-BAS algorithm.

## 1. Introduction

High resolution acquisition has been a hot topic in the field of synthetic aperture radar (SAR) systems and signal processing [1]. In addition, considerable studies have been performed on high-resolution SAR involved in both the large bandwidth [2,3,4,5,6] and sliding spotlight modes [1]. The conventional baseband azimuth scaling (BAS) algorithm [7] can work well with a sliding spotlight SAR signal with a carrier frequency that is much larger than the bandwidth. However, this algorithm is not suitable for the large bandwidth sliding spotlight SAR signals, because the approximation of the algorithm for sub-aperture data cannot decouple the sub-aperture signal in the large bandwidth mode. Targets deviating from the reference slant range defocus due to their inability to decouple the two dimensions’ high order phase binding caused by the large bandwidth mode. Additionally, the high resolution enabled by the large bandwidth sliding spotlight SAR is attracting increasing attention. Reference [8] first proposed the processing principle of the chirp scaling (CS) algorithm to solve the SAR signal in the large bandwidth mode and large squint angle mode. Zaugg of American Brigham Young University subsequently developed this principle and proposed a generalized high-order CS algorithm [9] that could compensate for the defocusing caused by the large bandwidth mode. At present, the generalized chirp scaling (GCS) algorithm has been a generalized signal processing method for SAR work in both large bandwidth and large squint modes.

The sliding spotlight SAR appropriately settles the conflicts between the high resolution and the large mapping range due to the fact that it can flexibly control the beam direction of the antenna [10]. For this reason, it has attracted many researchers to study sliding spotlight SAR more thoroughly and intensively. The requirements for data with high quality and accuracy have increased in the field of remote sensing in recent years. The higher accuracy imaging algorithms of high-resolution SAR are required by the increasing demands of high-resolution remote sensing data. Among sliding spotlight SAR imaging algorithms, many well-developed methods exist, such as the up-sampling imaging method, full-aperture processing method, and BAS algorithms. The up-sampling imaging method requires a large amount of data due to zero-padding processing [11]. Typically it acquires $B_{a\_total}/PRF$ (where $B_{a\_total}$ is the azimuth total bandwidth of the sliding spotlight SAR, and PRF is the pulse repetition frequency) times the data after zero-padding. The ω−k algorithm, which is introduced in the full-aperture processing method to solve the coupling problems along the range and azimuth dimensions, results in the algorithm time consumption [12]. Alberto Moreira of the German Aerospace Center (DLR) proposed an extended chirp scaling (ECS) algorithm that can solve the imaging problem of the sliding spotlight SAR in the antenna scanning modes [13]. Since then, the ECS algorithm has been the basic framework for multi-mode SAR imaging methods [13]. Pau Prats later developed the ECS algorithm and presented the BAS algorithm [7] to solve the imaging problem in the spotlight, Terrain Observation by Progressive Scans (TOPS), and sliding spotlight modes. In this method, the conventional CS algorithm is used to process the sub-aperture data of sliding spotlight SAR to eliminate the coupling between the range and azimuth dimensions successfully. After decoupling in the BAS algorithm, the Doppler rate of these sub-aperture data is adjusted again. After recombination of all sub-apertures, a de-rotation function and an azimuth compressed function are used to focus the sliding spotlight SAR data. Moreover, the experimental results of Terra SAR in the sliding spotlight and TOPS modes also prove the validity of the BAS algorithm.

With the increasing requirements for data accuracy in the remote sensing field, studies on the feasibility of high-resolution spaceborne/airborne SAR systems and imaging algorithms for high-resolution SAR are gaining more attention. However, the conditions of large bandwidth and low carrier frequency are not taken into consideration according to the BAS algorithm [14]. In other words, when $B_{r}/(2f_{0})>0.04$, the condition:

(1) $$f_{0}\gg \frac{B_{r}}{2}$$

is invalid, and the second-order approximation model of the BAS algorithm cannot address the high-order term, which can cause large phase errors and defocus the SAR images under the large bandwidth condition. The impact of the large bandwidth is involved in this paper in detail. In addition, taking the ideas of [8,9] about the processing of the large bandwidth SAR into account, this paper presents the method of the GCS-BAS algorithm for large bandwidth sliding spotlight SAR imaging. The proposed algorithm can improve the focus depth and accuracy of the processing results of the BAS algorithm for high-resolution signals from the large bandwidth sliding spotlight SAR.

The remainder of this paper is organized as follows. Section 2 analyzes the influence of the second-order approximation model in the conventional BAS algorithm on the phase of the signal from the large bandwidth sliding spotlight SAR. Section 3 presents the algorithm framework of the proposed GCS-BAS imaging algorithm and deduces the phase expressions and phase filters in each step of the proposed algorithm in detail. Finally, Section 4 compares the imaged results of the simulation by the conventional BAS algorithm, the back projection (BP) algorithm, and the GCS-BAS algorithm proposed in this paper.

## 2. Analysis of Model Error

**Figure 1:**
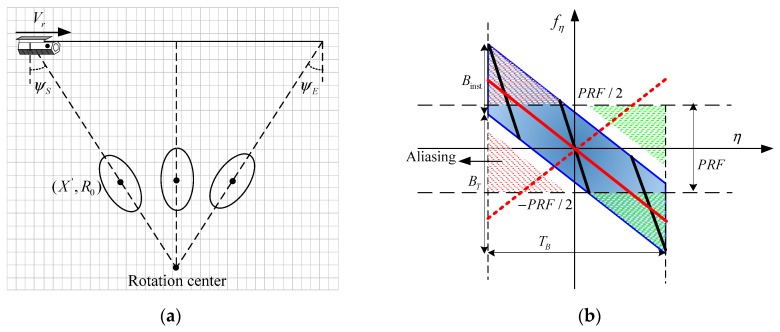
Schematic of the acquisition geometry for sliding spotlight spotlight synthetic aperture radar (SAR) (**a**) and time frequency distribution (TFD) of sliding spotlight SAR data (**b**). $\psi_{S}$ and $\psi_{E}$ represent the start and end scanning angles, respectively. PRF is the pulse repetition frequency. $B_{\mathrm{inst}}$ represents the instant azimuth bandwidth [16], and $B_{T}$ is the additional band-width resulting from the beam steering [16].

From Figure 1, the echo of the target in $\left(X^{'},R_{0}\right)$ demodulated to the baseband can be illustrated as:

(2) $$\begin{array}{c} s_{sliding\_spotlight}(\eta ,\tau )=W_{r}\left(\tau -\tau_{0}\right)\cdot W_{a}\left(\eta -\eta_{c}\right)\cdot \exp\left(j\pi K_{r}{\left(\tau -\tau_{0}\right)}^{2}\right)\cdot \\ \exp\left(-j2\pi f_{0}\tau_{0}\right)\cdot \mathrm{rect}\left(\frac{(V_{g}/V_{r})x-X^{'}}{L_{a}}\right) \end{array}$$

where τ is the fast time and η is the slow time. $\eta_{c}$ represents the moment of the Doppler centroid. $W_{r}\left(\cdot \right)$ and $W_{a}\left(\cdot \right)$ are the antenna patterns along the range and azimuth dimensions, respectively. $T_{r}$ is the pulse width. $K_{r}$ is the chirp rate of the transmit signal. $V_{g}$ and $V_{r}$ represent the velocities of beam steering on the ground and the radar, respectively. $\tau_{0}=\frac{2R\left(\eta \right)}{c}$ represents the delay of the target. R(η) is the range history of the target, expressed as $R\left(\eta \right)=\sqrt{R_{0}^{2}+V_{r}^{2}\eta^{2}}$. $R_{0}$ is the nearest range between the radar and the target. c is the speed of light. x is the trajectory of the radar. $L_{a}$ is the length of the synthetic aperture. rect(·) represents the illumination area of the beam [15]. A two dimensional (2D) fast Fourier transform (FFT) yields a signal in the 2D frequency domain:

(3) $$\theta_{2D}\left(f_{\tau },f_{\eta }\right)=-\frac{4\pi R_{0}f_{0}}{c}\sqrt{D\left(f_{\eta },V_{r}\right)+2\frac{f_{\tau }}{f_{0}}+\frac{f_{\tau }}{f_{0}^{2}}}-\frac{\pi f_{\tau }^{2}}{K_{r}}$$

where $D\left(f_{\eta },V_{r}\right)=\sqrt{1-\frac{c^{2}f_{\eta }^{2}}{4V_{r}^{2}f_{0}^{2}}}$, $f_{\eta }$ and $f_{\tau }$ are the azimuth frequency and range frequency, respectively. On the condition of $\frac{B_{r}}{2f_{0}}>0.04$ [9], the conventional CS is only able to compensate the high-order phase error of the reference range. However, it cannot resolve the high-order phase error caused by the large bandwidth that varies along the range. This problem leads to reduced precision for the secondary range compression (SRC) and range cell migration correction (RCMC). This degradation causes sub-aperture focusing performance that results in the deterioration of azimuth focusing later in the operation of azimuth scaling of the BAS. Therefore, the high-order phase error variation along the range should be taken into account to separate the coupling between the range and azimuth dimensions.

To illustrate the influence of the order-2 model approximation, a simulation is performed with the parameters listed in Table 1.

**Figure 2:**
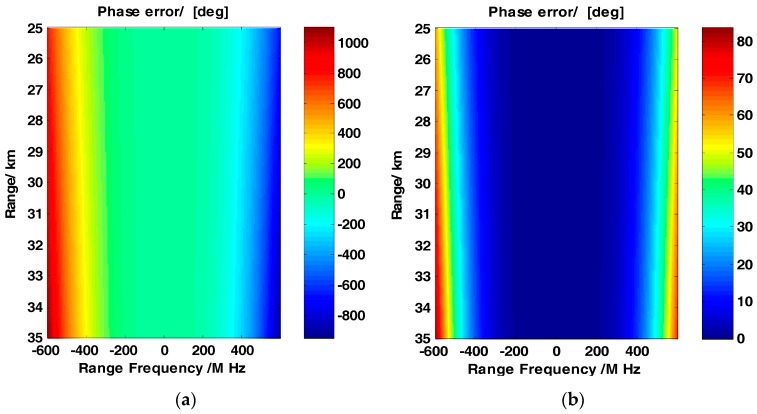

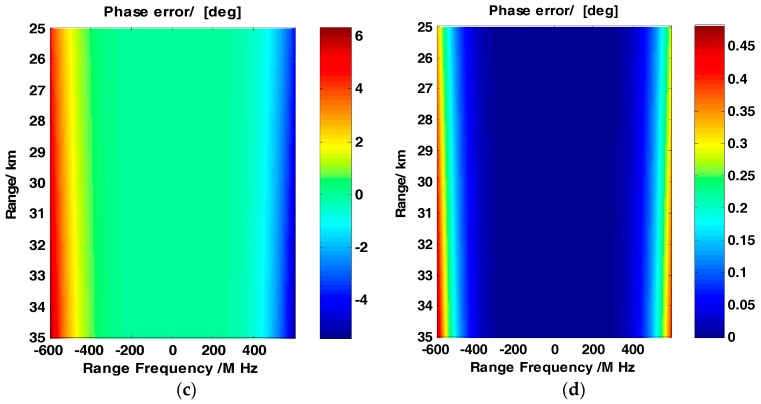
(**a**–**d**) shows the phase error of order-2–order-5 approximations, respectively. The color-bar represents the magnitude of the phase error by different colors and the horizontal and vertical axes represent the range frequency and range, respectively.

Figure 2a–d illustrates the phase error for different order approximations. The horizontal and vertical axes represent the range frequency and range, respectively. The color bar represents the magnitude of the phase error in different colors. Figure 2a illustrates that the phase error of the order-2 approximation is small for bandwidths between 400 and 600 MHz. This phase error can be neglected in the process of the sliding spotlight SAR. When the bandwidth is larger than 600 MHz, the phase error becomes large regardless of the reference range or edge range. The impact of the phase error is discussed in the previous paragraph. Figure 2b–d show the phase error of different order model approximations. The figures demonstrate that the phase error becomes lower as the order of the model increases. In Figure 2d, the phase error is reduced to 1 degree; this phase error is considered to have a small impact on the range compression and RCMC. High-order phase coupling should be considered in the processing of the large bandwidth sliding spotlight SAR. Each processing step for the large bandwidth sliding spotlight SAR is discussed below.

## 3. GCS-BAS

### 3.1. Procedures of GCS-BAS

**Figure 3:**
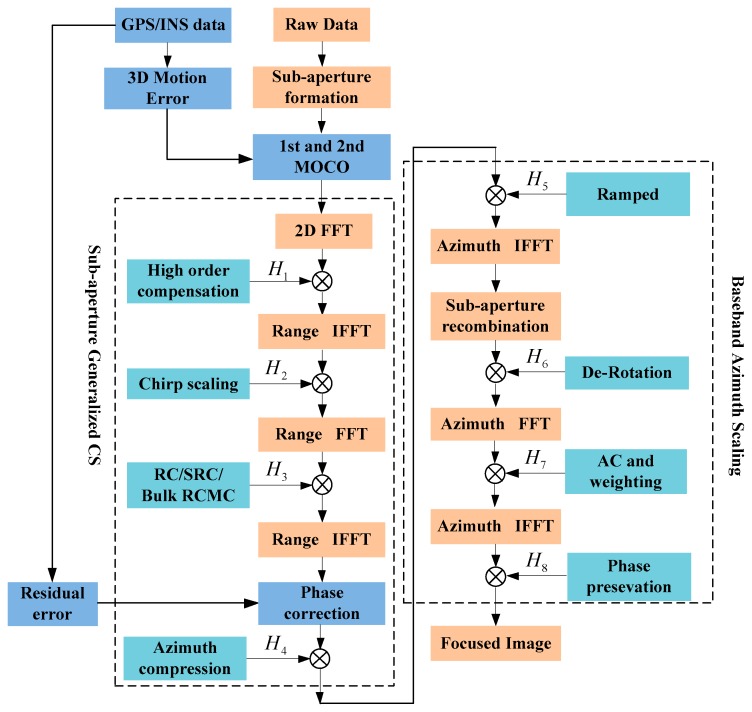
Blocks of proposed generalized chirp scaling-baseband azimuth scaling (GCS-BAS) for the large bandwidth sliding spotlight SAR image.

The blocks of processing procedures for GCS-BAS are shown in Figure 3, while the following text and Appendix A derive the signal of each step for GCS-BAS.

Reference [7] explains how to divide the raw data into the processed sub-apertures’ data. The steps are as follows:

Step 1: Considering the motion error of the platform, we adopt the method presented by [17] to eliminate the impact of motion error (readers can refer to [17]). This method has the advantage of eliminating the motion error on the basis of the platform trajectory measured by a precise device like global position system (GPS) or inertial navigation system (INS). The precise trajectory of the platform is easy to obtain in the simulation. Then the 1st and 2nd motion errors are compensated by the parameters of the 3D motion error which are extracted from the GPS/INS data [17]. 

Step 2: FFT is implemented to transfer the sliding spotlight SAR sub-aperture data to the 2D frequency domain. Then, the sliding spotlight SAR sub-aperture data in the 2D frequency domain are multiplied by the high-order compensation function;

Step 3: After high-order compensation, the sub-aperture data are transformed by inverse FFT (IFFT) along the range, and are then multiplied by a high-order CS function;

Step 4: After high-order CS, the sub-aperture data are transferred to the 2D frequency domain by FFT along the range. Then, the sub-aperture data are processed along the range with range compression (RC), SRC, and RCMC;

Step 5: The sub-aperture data are transferred into the range-Doppler domain by IFFT along the range. The phase error caused by the motion error is corrected by the residual error compensation which is calculated from the GPS/INS data [17]. It can be thought that the impact of the motion error is eliminated after the processing of the residual error correction [17]. Then, the data are multiplied by the azimuth compression function to achieve sub-aperture data imaging.

Steps 6–9: These steps of the BAS are described in [7] in detail. Functions of $H_{1}\sim H_{8}$ are explained in detail in the following text.

### 3.2. Theoretical Formulation

The following formulation is simplified with the form of the phase for the GCS-BAS algorithm which primarily processes phase functions. Equation (3) is expanded by a high-order Taylor series expansion.

(4) $$\begin{array}{cc} \theta_{2D} & =-\frac{4\pi R_{0}f_{0}}{c}D\left(f_{\eta }\right)-\frac{4\pi R_{0}}{cD\left(f_{\eta }\right)}f_{\tau }-\frac{4\pi R_{0}f_{0}}{c}\frac{\left(-1+D^{2}\left(f_{\eta }\right)\right)f_{\tau }^{2}}{2D^{3}\left(f_{\eta }\right)f_{0}^{2}}-\frac{\pi f_{\tau }^{2}}{K_{r}}\\ & -\frac{4\pi R_{0}f_{0}}{c}\sum_{i=3}^{n}\gamma_{i}f_{\tau }^{i} \end{array}$$

where $R_{0}$ is the slant range vector, and the coefficients $\gamma_{i}$ are discussed in [9]. We filter the sub-aperture raw data of the sliding spotlight and the large bandwidth SAR in the 2D frequency domain with the function:

(5) $$H_{1}\left(f_{\tau },f_{\eta }\right)=\exp\left[j\pi \sum_{i=3}^{n}\left(X_{i}+\frac{4R_{ref}f_{0}\gamma_{i}}{c}\right)f_{\tau }^{i}\right]$$

where $X_{i}$ is a high order turbulent item, which can simplify the GCS algorithm expressions, and its specific equation is discussed in Appendix A. The second item in $H_{1}\left(f_{\tau },f_{\eta }\right)$ is the compensation of a high order phase in the reference slant range that can minimize the range variation of a high order phase [18]. After high order phase compensation, the phase in the 2D frequency domain can be expressed by $\theta_{1}\left(f_{\tau },f_{\eta }\right)$:

(6) $$\theta_{1}\left(f_{\tau },f_{\eta }\right)=-\frac{4\pi R_{0}f_{0}}{c}D\left(f_{\eta }\right)-\frac{4\pi R_{0}}{cD\left(f_{\eta }\right)}f_{\tau }-\frac{\pi f_{\tau }^{2}}{K_{m}}+\pi \sum_{i=3}^{n}\left[X_{i}-\frac{4\left(R_{0}-R_{ref}\right)f_{0}}{c}\gamma_{i}\right]f_{\tau }^{i}$$

Then, IFFT is performed along the range for the sub-aperture SAR data after high order phase compensation. The solution of a stationary phase point can be calculated based on the principle of the stationary phase (POSP), $f_{\begin{array}{cc} \tau , & POSP \end{array}}=K_{m}\left(\tau -\tau_{d}\right)$. The processed sub-aperture data phase in the Range Doppler (RD) domain can be expressed by $\theta_{2}\left(\tau ,f_{\eta }\right)$:

(7) $$\begin{array}{cc} \theta_{2}\left(\tau ,f_{\eta }\right) & =-\frac{4\pi R_{0}f_{0}}{c}D\left(f_{\eta }\right)+\pi K_{m}{\left(\tau -\tau_{d}\right)}^{2}+\\ & \pi \sum_{i=3}^{n}\left[X_{i}-2f_{0}D\left(f_{\eta }\right)\gamma_{i}\Delta \tau \right]K_{m}^{i}{\left(\tau -\tau_{d}\right)}^{i} \end{array}$$

**Figure 4:**
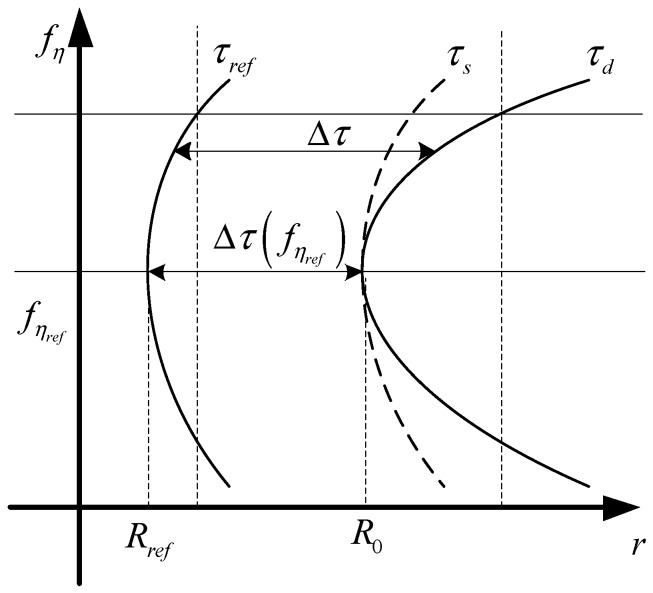
Chirp scaling for differential range cell migration correction.

Differential RCMC can be achieved by the method shown in Figure 4. The relationships between $\tau_{s}$, $\tau_{ref}$, $\tau_{d}$, $\Delta \tau \left(f_{\eta_{ref}}\right)$, and Δτ are explained in detail in Appendix A. The method for deducing the high order CS function is also discussed in Appendix A. The high order CS function [19] can be expressed by $H_{2}\left(\tau ,f_{\eta }\right)$:

(8) $$H_{2}\left(\tau ,f_{\eta }\right)=\exp\left[j\pi q_{2}{\left(\tau -\tau_{ref}\right)}^{2}+j\pi \sum_{i=3}^{n}q_{i}{\left(\tau -\tau_{ref}\right)}^{i}\right]$$

where the expressions of $q_{i}\left(i\geq 2\right)$ are explained in Appendix A. This step mainly uses the high order CS function to compensate for the influences of high order range cell migration (RCM) caused by the large bandwidth [20]. The phase of the sub-aperture data after processing high orders can be expressed by $\theta_{2}\left(\tau ,f_{\eta }\right)$:

(9) $$\begin{array}{cc} \theta_{2}\left(\tau ,f_{\eta }\right) & =-\frac{4\pi R_{0}f_{0}}{c}D\left(f_{\eta }\right)+\pi K_{m}{\left(\tau -\tau_{d}\right)}^{2}+\pi q_{2}{\left(\tau -\tau_{ref}\right)}^{2}+\pi \sum_{i=3}^{n}q_{i}{\left(\tau -\tau_{ref}\right)}^{i}\\ & +\pi \sum_{i=3}^{n}\left[X_{i}-2f_{0}D\left(f_{\eta }\right)\gamma_{i}\Delta \tau \right]K_{m}^{i}{\left(\tau -\tau_{d}\right)}^{i} \end{array}$$

Also $\theta_{2}\left(\tau ,f_{\eta }\right)$ can be expressed by the other form for the relationships between the coefficients $q_{i}\left(i\geq 2\right)$ and $X_{i}\left(i\geq 3\right)$:

(10) $$\begin{array}{cc} \theta_{2}\left(\tau ,f_{\eta }\right) & =-\frac{4\pi R_{0}f_{0}}{c}D\left(f_{\eta }\right)+\pi C_{0}\left(\Delta \tau \right)+\sum_{i=1}^{n}C_{i}{\left(\tau -\tau_{s}\right)}^{i}\\ & \approx -\frac{4\pi R_{0}f_{0}}{c}D\left(f_{\eta }\right)+\pi C_{0}\left(\Delta \tau \right)+\pi \frac{K_{f}}{\alpha }{\left(\tau -\tau_{s}\right)}^{2}\\ & +\sum_{i=3}^{n}\left(q_{i}+K_{f}^{i}X_{i}\right){\left(\tau -\tau_{s}\right)}^{i} \end{array}$$

The definitions of $C_{0}\left(\Delta \tau \right)$, α, $C_{i}$, and $K_{f}$ are provided in Appendix A. FFT is performed along the range dimension for the sub-aperture data with the phase of Equation (10) in the RD domain. Neglecting the impact of high order items (3 or higher) on the stationary phase point [12], we can obtain the solution of the stationary phase point by the POSP, $\tau_{POSP}=\frac{\alpha }{K_{f}}f_{\tau }+\tau_{s}$. The phase after FFT can be expressed by $\theta_{3}\left(f_{\tau },f_{\eta }\right)$:

(11) $$\begin{array}{cc} \theta_{3}\left(f_{\tau },f_{\eta }\right) & \approx -\frac{4\pi R_{0}f_{0}}{c}D\left(f_{\eta }\right)+\pi C_{0}\left(\Delta \tau \right)-\pi \frac{\alpha }{K_{f}}f_{\tau }^{2}-2\pi f_{\tau }\tau_{s}\\ & +\pi \sum_{i=3}^{n}\left(q_{i}+K_{f}^{i}X_{i}\right){\left(\frac{\alpha }{K_{f}}\right)}^{i}f_{\tau }^{i}\\ & =-\frac{4\pi R_{0}f_{0}}{c}D\left(f_{\eta }\right)+\pi C_{0}\left(\Delta \tau \right)-2\pi \tau_{ref}\left(1-\alpha \right)f_{\tau }-2\pi \alpha \tau_{d}f_{\tau }\\ & -\pi \frac{\alpha }{K_{f}}f_{\tau }^{2}+\pi \sum_{i=3}^{n}\left(q_{i}+K_{f}^{i}X_{i}\right){\left(\frac{\alpha }{K_{f}}\right)}^{i}f_{\tau }^{i} \end{array}$$

In Equation (11), the first term is the phase item related to the azimuth compression; the second term is the so-called residual video phase (RVP) [21]; the third term is the bulk RCM; the forth term is the linear phase, which is related to the target’s position along the range; the fifth and sixth terms are the phase that influence the accuracy of RC. $H_{3}\left(f_{\tau },f_{\eta }\right)$ is multiplied to achieve the RC, secondary RC and bulk RCMC.

(12) $$H_{3}\left(f_{\tau },f_{\eta }\right)=\exp\left\{j\left[2\pi \tau_{ref}\left(1-\alpha \right)f_{\tau }+\pi \frac{\alpha }{K_{f}}f_{\tau }^{2}-\pi \sum_{i=3}^{n}\left(q_{i}+K_{f}^{i}X_{i}\right){\left(\frac{\alpha }{K_{f}}\right)}^{i}f_{\tau }^{i}\right]\right\}$$

Filtered by $H_{3}\left(f_{\tau },f_{\eta }\right)$, the phase of the signal in the 2D frequency domain can be expressed as $\theta_{4}\left(f_{\tau },f_{\eta }\right)$:

(13) $$\theta_{4}\left(f_{\tau },f_{\eta }\right)=-\frac{4\pi R_{0}f_{0}}{c}D\left(f_{\eta }\right)+\pi C_{0}\left(\Delta \tau \right)-2\pi \alpha \tau_{d}f_{\tau }$$

The second term in Equation (13) is the RVP that must be eliminated in the RD domain. A range IFFT using POSP leads to the following phase:

(14) $$\theta_{4}\left(\tau ,f_{\eta }\right)=-\frac{4\pi R_{0}f_{0}}{c}D\left(f_{\eta }\right)+\pi C_{0}\left(\Delta \tau \right)$$

Thus, we can obtain the azimuth compression function based on Equation (14):

(15) $$H_{4}\left(\tau ,f_{\eta }\right)=\exp\left\{j\pi \left[\frac{4\pi R_{0}f_{0}}{c}\left[D\left(f_{\eta }\right)-1\right]-\pi C_{0}\left(\Delta \tau \right)\right]\right\}$$

Then, the signal in the RD domain can be expressed as:

(16) $$S_{4}\left(\tau ,f_{\eta }\right)=\omega_{r}\left(\tau -\frac{2R_{0}}{c}\right)\cdot W_{a}\left(f_{\eta_{c}}\right)\cdot \mathrm{sinc}\left(\tau -\frac{2R_{0}}{c}\right)\cdot \exp\left(-j\frac{4\pi R_{0}f_{0}}{c}\right)$$

After being processed by $H_{4}\left(\tau ,f_{\eta }\right)$, the phase coupling between the range and azimuth caused by the large bandwidth can be eliminated. If the coupling between the range and azimuth dimensions is not eliminated clearly, it leads to the unmatched Doppler rate in the following BAS algorithm’s azimuth scaling. This also results in defocusing in the azimuth dimension. The following operations are similar to the BAS algorithm’s azimuth scaling and hence are described only briefly here.

After azimuth compression for the sub-aperture data, a purely quadratic phase shape is added using:

(17) $$H_{5}\left(\tau ,f_{\eta }\right)=\exp\left(-j2\pi f_{\eta }\eta_{v}\left(R_{0}\right)\right)\exp\left(-j\frac{\pi }{K_{scl}\left(R_{0}\right)}f_{\eta }^{2}\right)$$

$K_{scl}\left(R_{0}\right)$ is the Doppler rate factor, which is related to the velocity $V_{r}$ and the range vector of $R_{scl}\left(R_{0}\right)$.

(18) $$K_{scl}\left(R_{0}\right)=-\frac{2V_{r}^{2}}{\lambda R_{scl}\left(R_{0}\right)}$$

**Figure 5:**
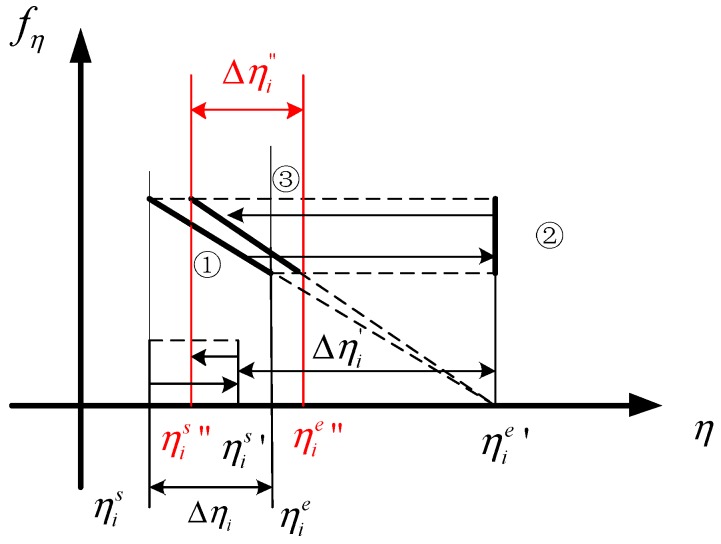
Variation of time-frequency distribution (TFD).

The factor is different from the one in Equation (15) that causes the extension along the azimuth dimension after azimuth scaling. The time shift $\eta_{v}\left(R_{0}\right)$ with a linear phase ramp is introduced to minimize the extension of azimuth dimension, whose expression is given in [15]. An azimuth IFFT is performed on the sub-aperture data after azimuth scaling. The shift and recombination operations need to be done while the sub-apertures are processed by $H_{1}\sim H_{5}$. The shift and recombination operations in Figure 5, as well as the meaning of variances, are discussed well in [22].

However, the total azimuth bandwidth still exceeds the PRF after all sub-apertures are assembled. Therefore a demodulation operation similar to the two-step algorithm [23] is carried out to move the signal to the azimuth baseband using the demodulation function:

(19) $$H_{6}\left(\tau ,\eta \right)=\exp\left(-j\pi K_{rot}\left(R_{0}\right)\eta^{2}\right)$$

(20) $$K_{rot}\left(R_{0}\right)=-\frac{2V_{r}^{2}}{\lambda R_{rot}\left(R_{0}\right)}$$

**Figure 6:**
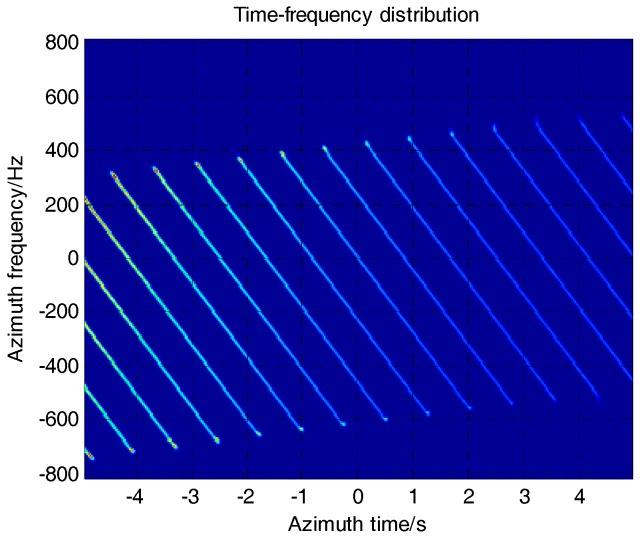
TFD of SAR data after azimuth derotation.

$R_{scl}\left(R_{0}\right)$ in Equation (18) and $R_{rot}\left(R_{0}\right)$ in Equation (20) are described in detail in [7]. The TFD after azimuth derotation is shown in Figure 6.

After filtered by $H_{6}\left(\tau ,\eta \right)$, the sliding spotlight SAR azimuth time sampling and image sampling vary with the following equations [13]:

(21) $$\begin{array}{l} \frac{1}{PRF_{0}}=\frac{1}{PRF}\cdot \left(1-\frac{R_{scl0}}{R_{rot0}}\right)\\ \Delta x_{final}=\Delta x_{orig}\left(1-\frac{R_{scl}\left(R_{0}\right)}{R_{rot}\left(R_{0}\right)}\right) \end{array}$$

where $PRF_{0}$ is the new azimuth sampling, $\Delta x_{final}$ is the final image sampling, and $\Delta x_{orig}$ is provided in [13], and the effective chirp rate has been changed after azimuth scaling and azimuth derotation:

(22) $$K_{eff}\left(R_{0}\right)=K_{scl}\left(R_{0}\right)-K_{rot}\left(R_{0}\right)$$

**Figure 7:**
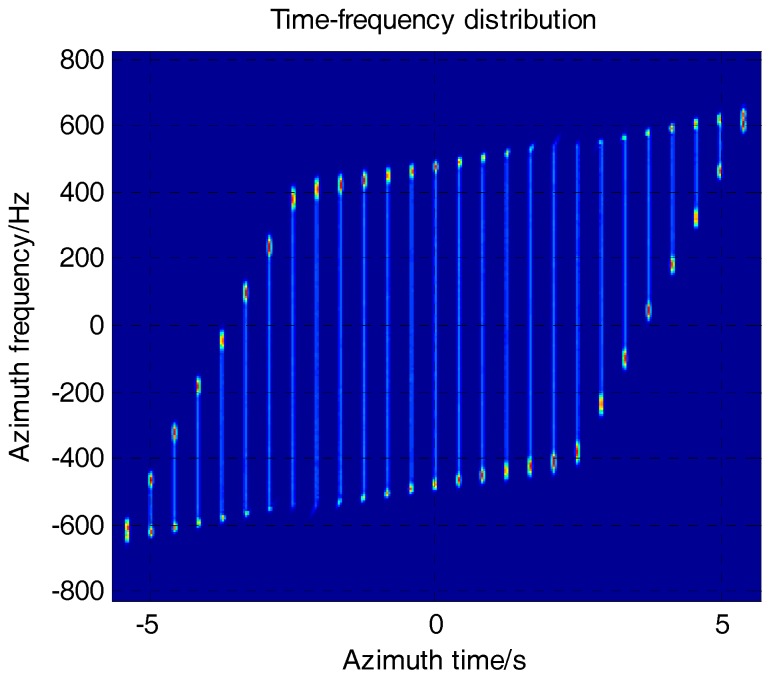
TFD of SAR data after phase preservation.

An azimuth FFT results in the sliding spotlight data in the RD domain. The azimuth match filter is as following:

(23) $$H_{7}\left(\tau ,f_{\eta }\right)=W_{a}\left(f_{\eta }\right)\cdot \exp\left(j\frac{\pi }{K_{eff}\left(R_{0}\right)}f_{\eta }^{2}\right)\begin{array}{cc} , & -\frac{PRF}{2}+f_{dc}<f_{\eta } \end{array}<\frac{PRF}{2}+f_{dc}$$

where $f_{dc}$ is the mean Doppler centroid of the whole data acquisition, and $W_{a}\left(f_{\eta }\right)$ is a weighting function which can suppress the side-lobe. An IFFT on the data after being filtered by the function $H_{7}\left(\tau ,f_{\eta }\right)$ yields the focused data. For phase preservation purposes, the data should be multiplied by the function:

(24) $$H_{8}\left(\tau ,\eta \right)=\exp\left(j\pi K_{t}\left(R_{0}\right){\left(1-\frac{r_{scl0}}{r_{rot0}}\right)}^{2}\eta^{2}\right)$$

where $K_{t}\left(R_{0}\right)=-\frac{2V_{r}^{2}}{\lambda \left(r_{rot}\left(R_{0}\right)-r_{scl}\left(R_{0}\right)\right)}$. The proposed function achieves the phase preservation efficiently without interpolation. The TFD after phase preservation is shown in Figure 7.

## 4. Experimental Results and Discussion

In this section, all experiments are carried out by a PC with an i7 Dual-Core CPU of 3.3 GHz and 32 GB memory. To verify the validity of the GCS-BAS algorithm, a simulation is performed with the parameters listed in Table 2.

**Figure 8:**
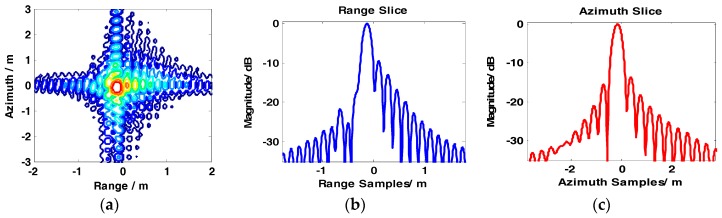

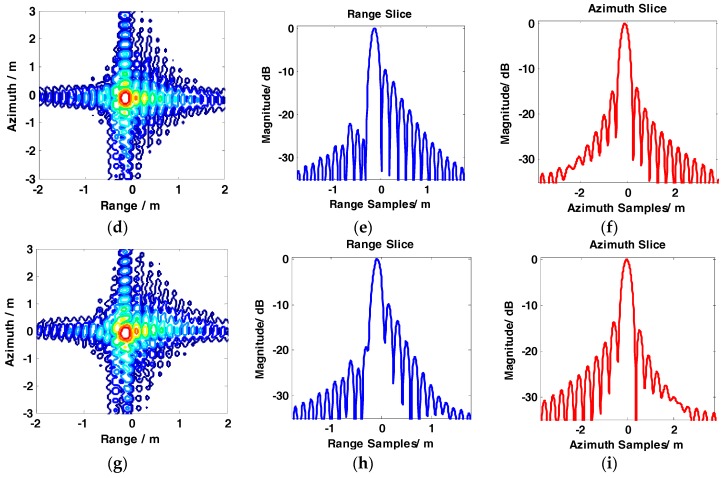
Imaging results of baseband azimuth scaling (BAS). (**a**–**c**) show contour, range slice, and azimuth slice of the target in the far range, respectively. (**d**–**f**) show contour, range slice, and azimuth slice of the target near the mid-range, respectively. (**g**–**i**) show contour, range slice, and azimuth slice of the target in the near range, respectively.

**Figure 9:**
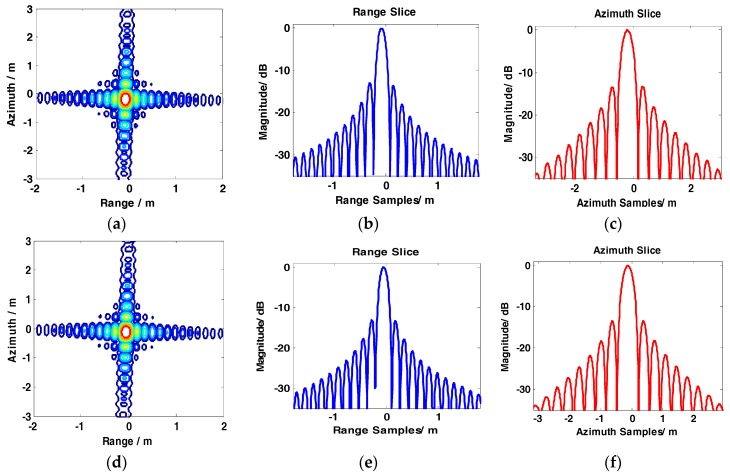

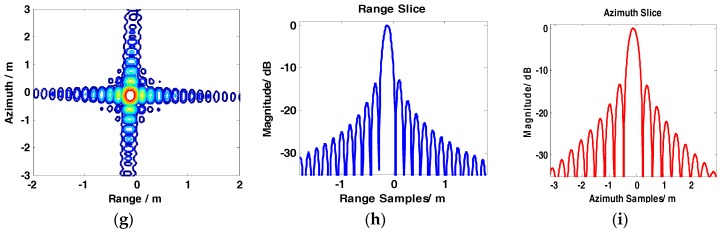
Imaging results of GCS-BAS. (**a**–**c**) show contour, range slice, and azimuth slice of the target in the far range, respectively. (**d**–**f**) show contour, range slice, and azimuth slice of the target near the mid-range, respectively. (**g**–**i**) show contour, range slice, and azimuth slice of the target in the near range, respectively.

**Figure 10:**
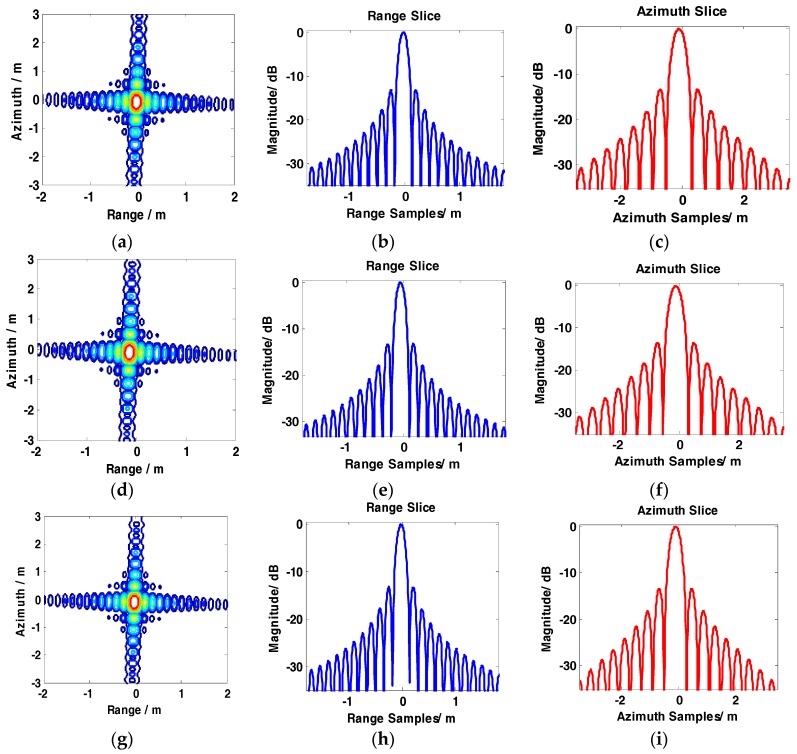
Imaging results of the full-aperture imaging algorithm with ω−k process kernel. (**a**–**c**) show contour, range slice, and azimuth slice of the target near the far range, respectively. (**d**–**f**) show contour, range slice, and azimuth slice of the target in the mid-range, respectively. (**g**–**i**) show contour, range slice, and azimuth slice of the target in the near range, respectively.

**Figure 11:**
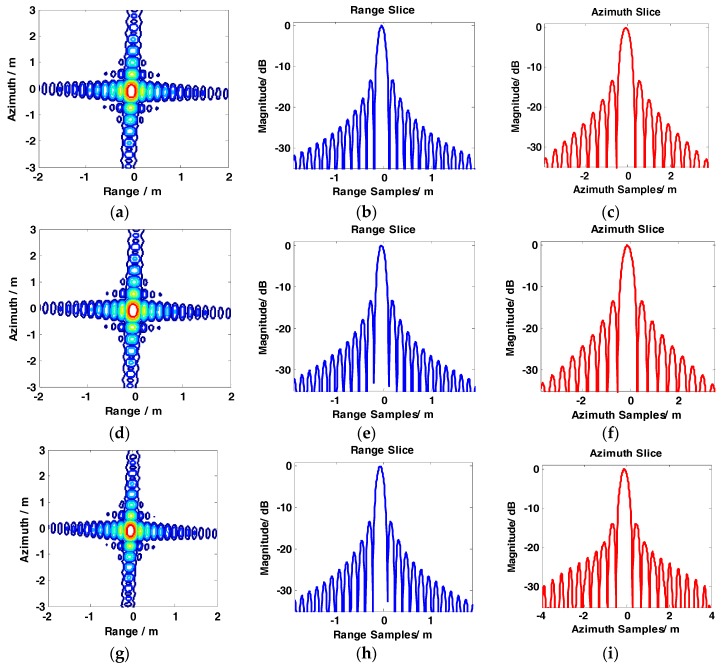
Imaging results of back-projection (BP). (**a**–**c**) show contour, range slice, and azimuth slice of the target near the far range, respectively. (**d**–**f**) show contour, range slice, and azimuth slice of the target in the mid-range, respectively. (**g**–**i**) show contour, range slice, and azimuth slice of the target in the near range, respectively.

The results of the conventional BAS, GCS-BAS, BP, and full-aperture imaging algorithms are presented to compare the focusing performance of the conventional BAS algorithm and GCS-BAS algorithm. The results of the four algorithms are shown in Figure 8, Figure 9, Figure 10 and Figure 11.

This paper analyses the focusing performance of the three algorithms based on such indices as the peak side-lobe ratio (PSLR), integrated side-lobe ratio (ISLR), and resolution (Res). The indices of target focusing for the four algorithms are shown in Table 3.

Figure 8 and Table 3 illustrate that the conventional BAS algorithm causes deterioration of the target image when the system is in the large bandwidth mode, such as with the parameters shown in Table 2. The PSLR, ISLR, and resolution degrade considerably in the two dimensions, particularly along the range edges. Degradation is also observed along the azimuth dimension. Figure 9 shows the results obtained by the GCS-BAS algorithm. The contours for different targets in Figure 9a,d,g, the range slices in Figure 9b,e,h, and the azimuth slices in Figure 9c,f,i show that the GCS-BAS algorithm is able to process the large bandwidth sliding spotlight SAR data whether what the target is located at the edges or in the center. Figure 10 and Table 3 illustrate that the focus indexes of the GCS-BAS algorithm are notably better than the results processed by the conventional BAS algorithm, which performs in a similar manner as the BP algorithm. Table 4 shows the statistical properties of targets at the azimuth edge. The standard deviations indicate that the full-aperture imaging algorithm with the ω−k processing kernel has a focusing capability which approaches GCS-BAS. The results of Figure 9 and Figure 10 and Table 4 show that the former two imaging algorithms have imaging capabilities which are closer to BP.

However, the four algorithms discussed in this paper has different computational burdens. Suppose that the number of pixels in the range direction and azimuth direction is $N_{rg}$ and $N_{az}$, respectively. Table 5 shows the computational burden of the BAS, GCS-BAS, BP, and full-aperture imaging algorithms with the ω−k processing kernel.

$M_{\ker}$ is the Stolt interpolation length of the ω−k processing kernel. To maintain a high focusing accuracy, $M_{\ker}$ is always greater than 16.

**Figure 12:**
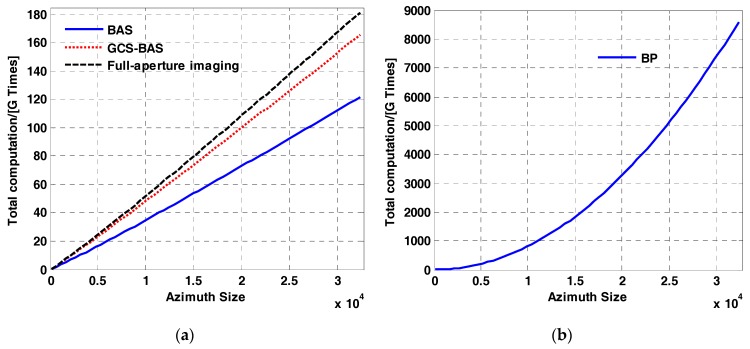
Computational burden of the four algorithms. (**a**) shows the computational burden with respect to azimuth size, with $N_{rg}=8192$, for BAS (solid blue), GCS-BAS (dotted red), and full-aperture imaging (dashed black). (**b**) shows the computational burden of BP with respect to azimuth size, with $N_{rg}=8192$.

Figure 12 illustrates the different computational burdens of the four algorithms with different $N_{az}$ and a constant $N_{rg}$. It is easy to conclude that the computational burden of BP is much heavier than that of the other three algorithms. The computational burden of the full-aperture imaging algorithm with the ω−k processing kernel is slightly heavier than that of BAS-GCS. Table 5 illustrates that the computational burden of the BP and full-aperture imaging algorithm with ω−k processing kernel are much larger than that of GCS-BAS. The Stolt interpolation in the ω−k processing kernel is much more time consuming than the common multiplication operation [24] in BAS or GCS-BAS. Hence, compared with the other three algorithms discussed in this paper, the GCS-BAS has the advantages of computational efficiency and focusing accuracy in the processing of the large bandwidth sliding spotlight SAR.

For a more intuitive understanding of the computational efficiency of the four algorithms, we process the data on the parameters shown in Table 2 by a computer with an i7 Dual-Core CPU of 3.3 GHz. The size of the raw data is $\begin{array}{ccc} N_{rg}\left(=40000\right) & \times & N_{az} \end{array}\left(=24000\right)$. The algorithms’ time consumption is shown in Table 6.

## 5. Conclusions 

The large bandwidth sliding spotlight SAR critically couples between the range and azimuth dimensions due to its large bandwidth relative to the carrier frequency. Its azimuth spectrum is also aliased for its antenna beam steering in velocity orientation, which causes the total azimuth bandwidth to be larger than that of PRF. This paper presents a method called the GCS-BAS algorithm to solve the coupling problem of the large bandwidth sliding spotlight SAR. The algorithm has a flexibility to choose the compensation order for the large bandwidth sliding spotlight SAR based on the ratio $f_{0}/B_{r}$. Compared with the up-sampling algorithm and the full-aperture processing algorithm, the GCS-BAS algorithm processes the large bandwidth sliding spotlight SAR data more efficiently without zero-padding or interpolation. The simulation and real data processing indicate that the algorithm is able to solve the phase coupling problem caused by a large bandwidth. Higher precision and accuracy can be achieved for the large bandwidth sliding spotlight SAR data compared to the conventional BAS algorithm. It is also more efficient than the full-aperture imaging algorithm with the ω−k processing kernel or the BP algorithm at the same focusing depth and accuracy. The simulation of the former four algorithms indicates that a fine focus can be achieved for the large bandwidth sliding spotlight data by the GCS-BAS algorithm, while an appropriate trade-off can be obtained between computational efficiency and imaging accuracy. 

## Figures and Tables

**Table 1:** Parameters for the model phase error.

Parameter	Value
Carrier frequency/$f_{0}$	8 GHz
Bandwidth/$B_{r}$	1 GHz
Reference range/$R_{ref}$	30 km
Pulse repetition frequency/PRF	600 Hz
Velocity of radar/$V_{r}$	240 m/s
Pulse width/$T_{r}$	40 μs

**Table 2:** Parameters for sliding spotlight SAR.

Parameter	Value
Carrier frequency/$f_{0}$	8 GHz
Bandwidth/$B_{r}$	1 GHz
Scaling range/$R_{ref}$	30 km
Pulse repetition frequency/PRF	600 Hz
Velocity of platform/$V_{r}$	240 m/s
Pulse width/$T_{r}$	40 μs
Image swath	5 km × 5 km
Factor of sliding spotlight/M	0.4

**Table 3:** Performance of the three algorithm imaging results.

Target Position	Dimension	Index	BAS	GCS-BAS	Full-Aperture Imaging	BP
Far range	Azimuth	PSLR/dB	−13.6593	**−13.3273**	−13.3364	−13.4676
		ISLR/dB	−12.4162	**−10.7198**	−10.6901	−10.6779
		Res/m	0.2490	**0.2371**	0.2365	0.2359
	Range	PSLR/dB	−9.7625	**−12.8918**	−13.0021	−13.2873
		ISLR/dB	−6.2740	**−10.1871**	−10.0817	−9.9379
		Res/m	0.1379	**0.1328**	0.1328	0.1328
Mid-range	Azimuth	PSLR/dB	−14.0884	**−13.4163**	−13.3701	−13.6043
		ISLR/dB	−11.7920	**−10.5783**	−10.5001	−10.1517
		Res/m	0.2321	**0.2229**	0.2203	0.2191
	Range	PSLR/dB	−9.5632	**−13.2104**	−13.2498	−13.2759
		ISLR/dB	−5.6929	**−9.9811**	−9.98758	−9.9629
		Res/m	0.1359	**0.1328**	0.1328	0.1328
Near range	Azimuth	PSLR/dB	−14.8360	**−13.2982**	−13.3125	−13.6205
		ISLR/dB	−10.9848	**−10.3992**	−10.0126	−9.9223
		Res/m	0.2371	**0.2234**	0.2190	0.2148
	Range	PSLR/dB	−9.4085	**−12.9537**	−13.0529	−13.2856
		ISLR/dB	−5.6916	**−9.6545**	−9.6581	−9.9877
		Res/m	0.1395	**0.1328**	0.1328	0.1328

**Table 4:** The statistical properties of targets at the azimuth edge.

Dimension	Index		BAS	GCS-BAS	Full-Aperture Imaging	BP
Range	Res	Mean/m	0.1351	0.1327	0.1328	0.1324
		**Standard deviation**	**0.0090**	**0.0010**	**0.0012**	**0.0002**
	PSLR	Mean/dB	−10.8216	−13.0939	−13.2842	−13.2889
		**Standard deviation**	**7.4778**	**0.5546**	**0.1766**	**0.0558**
	ISLR	Mean/dB	−7.4356	−10.0103	−9.9508	−9.8435
		**Standard deviation**	**6.6592**	**1.2482**	**1.2809**	**0.7351**
Azimuth	Res	Mean/m	0.2423	0.2348	0.2354	0.2348
		**Standard deviation**	**0.0238**	**0.0004**	**0.0018**	**0.0004**
	PSLR	Mean/dB	−13.4884	−13.3351	−13.3710	−13.3012
		**Standard deviation**	**0.7070**	**0.1968**	**0.3371**	**0.0948**
	ISLR	Mean/dB	−11.1393	−10.6051	−10.9491	−10.5896
		**Standard deviation**	**5.0295**	**3.0250**	**2.7883**	**2.6693**

**Table 5:** The comparison of algorithms’ computational burden.

Algorithms	Computational Burden
BAS	$29N_{rg}N_{az}+20N_{rg}N_{az}\log_{2}\left(N_{az}\right)+10N_{rg}N_{az}\log_{2}\left(N_{rg}\right)$
GCS-BAS	$64N_{rg}N_{az}+20N_{rg}N_{az}\log_{2}\left(N_{az}\right)+20N_{rg}N_{az}\log_{2}\left(N_{rg}\right)$
Full-aperture imaging	$\left(4M_{\ker}+40\right)N_{rg}N_{az}+30N_{rg}N_{az}\log_{2}\left(N_{az}\right)+10N_{rg}N_{az}\log_{2}\left(N_{rg}\right)$
BP	$N_{rg}N_{az}^{2}$

**Table 6:** Time consumption of the algorithms.

Algorithms	BAS	GCS-BAS	Full-Aperture Imaging	BP
Time consumption	1153.1293 s	1405.0256 s	3685.2732 s	160,484.2220 s

## Appendix A

This appendix is intended to help understand the different steps of sub-aperture processing of GCS-BAS. Expanding Equation (3) with the Tyler principle and the sub-aperture data with the phase of Equation (4) in the 2D frequency domain can be described as:

(A1) $$S_{1}\left(f_{\tau },f_{\eta }\right)=W_{r}\left(f_{\tau }\right)W_{a}\left(f_{\eta }\right)\exp\left(j\theta_{2D}\right)$$

$\theta_{2D}$ is described in Equation (4). After the high order phase compensation by $H_{1}\left(f_{\tau },f_{\eta }\right)$, the signal in the 2D domain can be expressed as:

(A2) $$S_{1}\left(f_{\tau },f_{\eta }\right)=W_{r}\left(f_{\tau }\right)W_{a}\left(f_{\eta }\right)\exp\left(j\theta_{1}\left(f_{\tau },f_{\eta }\right)\right)$$

$\theta_{1}\left(f_{\tau },f_{\eta }\right)$ is described in Equation (6). A range IFFT results in the signal in the RD domain:

(A3) $$S_{1}\left(\tau ,f_{\eta }\right)=w_{r}\left(\tau \right)W_{a}\left(f_{\eta }\right)\exp\left(j\theta_{2}\left(\tau ,f_{\eta }\right)\right)$$

$\theta_{2}\left(\tau ,f_{\eta }\right)$ is discussed in Equation (7). The SRC factor $K_{m}$ and time in the RD domain are:

(A4) $$K_{m}=\frac{K_{r}}{1-K_{r}\frac{cR_{0}f_{\eta }^{2}}{2V_{r}^{2}f_{0}^{3}D{\left(f_{\eta }\right)}^{3}}}$$

(A5) $$\begin{array}{l} \tau_{d}=\frac{2R_{0}}{cD\left(f_{\eta }\right)}\\ \tau_{ref}=\frac{2R_{ref}}{cD\left(f_{\eta }\right)}\\ \Delta \tau =\tau_{d}-\tau_{ref} \end{array}$$

The following factors can be deduced from Equations (A4) and (A5):

(A6) $$\begin{array}{l} K_{s}=\frac{c^{2}f_{\eta }^{2}}{4v^{2}f_{0}^{3}D^{2}}\\ K_{f}=\frac{K_{r}}{1-K_{r}\tau_{ref}K_{s}} \end{array}$$

The time in the RD domain is represented as the total RCM:

(A7) $$\begin{array}{cc} RCM_{total} & =\frac{2R_{0}}{cD\left(f_{\eta }\right)}-\frac{2R_{0}}{cD\left(f_{\eta_{ref}}\right)}=\frac{2R_{0}}{cD\left(f_{\eta }\right)}\left(1-\frac{D\left(f_{\eta }\right)}{D\left(f_{\eta_{ref}}\right)}\right)\\ & =\tau_{d}\left(1-\alpha \right) \end{array}$$

We define $\alpha =\frac{D\left(f_{\eta }\right)}{D\left(f_{\eta_{ref}}\right)}$. The time delay of the bulk RCM can be expressed as:

(A8) $$\begin{array}{cc} RCM_{bulk} & =\frac{2R_{ref}}{cD\left(f_{\eta },V_{r_{ref}}\right)}-\frac{2R_{ref}}{cD\left(f_{\eta_{ref}},V_{r_{ref}}\right)}=\frac{2R_{ref}}{cD\left(f_{\eta },V_{r_{ref}}\right)}\left(1-\frac{D\left(f_{\eta },V_{r_{ref}}\right)}{D\left(f_{\eta_{ref}},V_{r_{ref}}\right)}\right)\\ & \approx \tau_{ref}-\alpha \tau_{ref} \end{array}$$

The following equation can be obtained from Figure 4:

(A9) $$\begin{array}{cc} \Delta \tau \left(f_{\eta_{ref}}\right) & =\frac{2R_{0}}{cD\left(f_{\eta_{ref}}\right)}-\frac{2R_{ref}}{cD\left(f_{\eta_{ref}}\right)}=\frac{D\left(f_{\eta }\right)}{D\left(f_{\eta_{ref}}\right)}\left(\frac{2R_{0}}{cD\left(f_{\eta }\right)}-\frac{2R_{ref}}{cD\left(f_{\eta }\right)}\right)\\ & =\alpha \Delta \tau \end{array}$$

(A10) $$\tau_{s}=\tau_{ref}+\Delta \tau \left(f_{\eta_{ref}}\right)=\tau_{ref}+\alpha \Delta \tau =\tau_{d}-\Delta \tau \left(1-\alpha \right)$$

The differential RCM can be expressed as:

(A11) $$\begin{array}{cc} RCM_{diff} & =RCM_{total}-RCM_{bulk}=\tau_{d}\left(1-\alpha \right)-\tau_{ref}\left(1-\alpha \right)\\ & =\left(1-\alpha \right)\left(\tau_{d}-\tau_{ref}\right)=\left(1-\alpha \right)\Delta \tau \end{array}$$

Differential RCMC is achieved by the CS operation. From the phase perspective, the CS realizes the addition of Equations (7) and (8). Expand Equation (9) at $\tau =\tau_{s}$, and the following equation is obtained:

(A12) $$\begin{array}{cc} \theta_{2}\left(\tau ,f_{\eta }\right) & =-\frac{4\pi R_{0}f_{0}}{c}D\left(f_{\eta }\right)+\pi K_{m}{\left(\left(\tau -\tau_{s}\right)-\left(1-\alpha \right)\Delta \tau \right)}^{2}\\ & +\pi \sum_{i=3}^{n}\left[X_{i}-2f_{0}D\left(f_{\eta }\right)\gamma_{i}\Delta \tau \right]K_{m}^{i}{\left(\left(\tau -\tau_{s}\right)-\left(1-\alpha \right)\Delta \tau \right)}^{i}\\ & +\pi q_{2}{\left(\left(\tau -\tau_{s}\right)+\alpha \Delta \tau \right)}^{2}+\pi \sum_{i=3}^{n}q_{i}{\left(\left(\tau -\tau_{s}\right)+\alpha \Delta \tau \right)}^{i}\\ & =-\frac{4\pi R_{0}f_{0}}{c}D\left(f_{\eta }\right)+\pi C_{0}\left(\Delta \tau \right)+\sum_{i=1}^{n}C_{i}{\left(\tau -\tau_{s}\right)}^{i} \end{array}$$

$C_{0}\left(\Delta \tau \right)$ is the polynomial of Δτ, and it can be expressed as:

(A13) $$\begin{array}{cc} C_{0}\left(\Delta \tau \right) & =\left(\alpha^{2}q_{2}+{\left(\alpha -1\right)}^{2}K_{m}\right)\Delta \tau^{2}+\left(\alpha^{3}q_{3}+{\left(\alpha -1\right)}^{3}K_{m}^{3}X_{3}\right)\Delta \tau^{3}\\ & +\sum_{i=4}^{n}\left(\alpha^{i}q_{i}+{\left(\alpha -1\right)}^{i}K_{m}^{i}X_{i}-2D\left(f_{\eta }\right)f_{0}\gamma_{i-1}{\left(\alpha -1\right)}^{i-1}K_{m}^{i-1}\right)\Delta \tau^{i} \end{array}$$

The coefficients of $\left(\tau -\tau_{s}\right)$ are calculated from Equation (A12) (this paper only calculates to $C_{3}$ due to space restraints).

(A14) $$\begin{array}{cc} C_{1} & =2\left[\left(-1+\alpha \right)K_{m}+\alpha q_{2}\right]\Delta \tau +\left[3\alpha^{2}q_{3}+3{\left(-1+\alpha \right)}^{2}K_{m}^{3}X_{3}\right]\Delta \tau^{2}+\sum_{i=3}^{n}C_{1_{i}}\Delta \tau^{i}\\ C_{2} & =q_{2}+K_{m}+3\left[\alpha q_{3}+\left(-1+\alpha \right)K_{m}^{3}X_{3}\right]\Delta \tau \\ & +6\left[\alpha^{2}q_{4}+{\left(-1+\alpha \right)}^{2}K_{m}^{4}X_{4}-\left(-1+\alpha \right)K_{m}^{3}D\left(f_{\eta }\right)f_{0}\gamma_{3}\right]\Delta \tau^{2}+\sum_{i=3}^{n}C_{2_{i}}\Delta \tau^{i}\\ C_{3} & =q_{3}+K_{m}^{3}X_{3}+\left[4\alpha q_{4}+4K_{m}^{4}X_{4}(-1+\alpha )-2K_{m}^{3}D\left(f_{\eta }\right)f_{0}\gamma_{3}\right]\Delta \tau \\ & +\left[10\alpha^{2}q_{5}+10{\left(-1+\alpha \right)}^{2}K_{m}^{5}X_{5}-8(-1+\alpha )K_{m}^{4}D\left(f_{\eta }\right)f_{0}\gamma_{4}\right]\Delta \tau^{2}\\ & +\sum_{i=3}^{n}C_{3_{i}}\Delta \tau^{i}\\ & \ldots \ldots \end{array}$$

Equation (A4) is expanded at Δτ=0 with the Tyler principle. Then Equation (A4) can be expressed as:

(A15) $$K_{m}=\frac{K_{f}}{1-K_{f}\Delta \tau K_{s}}\approx K_{f}+K_{s}K_{f}^{2}\Delta \tau +K_{s}^{2}K_{f}^{3}\Delta \tau^{2}$$

Based on Equations (A15) and (A14), we can obtain the solutions of $q_{i}\left(i\geq 2\right)$ and $X_{i}\left(i\geq 3\right)$ for setting the first- and second-order coefficients of Δτ to zero.

(A16) $$\left\{ \begin{array}{c} q_{2}=\frac{K_{f}\left(1-\alpha \right)}{\alpha }\\ q_{3}=\frac{(1-\alpha )K_{f}^{2}K_{s}}{3\alpha }\\ X_{3}=\frac{(-2+\alpha )K_{s}}{3(-1+\alpha )K_{f}}\\ q_{4}=-\frac{K_{f}^{3}(2K_{s}^{2}+9(-1+\alpha )K_{f}K_{s}X_{3}-6D\left(f_{\eta }\right)(-1+\alpha )f_{0}\gamma_{3})}{12\alpha }\\ X_{4}=\frac{2K_{s}^{2}+9(-2+\alpha )K_{f}K_{s}X_{3}-6D\left(f_{\eta }\right)(-2+\alpha )f_{0}\gamma_{3}}{12(-1+\alpha )K_{f}}\\ \ldots \ldots \end{array}\right.$$

After the CS operation, the signal in the RD domain can be expressed as:

(A17) $$S_{2}\left(\tau ,f_{\eta }\right)=w_{r}\left(\tau \right)W_{a}\left(f_{\eta }\right)\exp\left(j\theta_{2}\left(\tau ,f_{\eta }\right)\right)$$

$\theta_{2}\left(\tau ,f_{\eta }\right)$ is described in Equation (10). A range FFT is used for the transformation into the 2D frequency domain. Neglecting the impact of a third order higher-order item, the result of the stationary phase point is $\tau_{POSP}=\frac{\alpha }{K_{f}}f_{\tau }+\tau_{s}$. The signal can be expressed as:

(A18) $$S_{3}\left(f_{\tau },f_{\eta }\right)=W_{r}\left(f_{\tau }\right)W_{a}\left(f_{\eta }\right)\exp\left[j\pi \theta_{3}\left(f_{\tau },f_{\eta }\right)\right]$$

$\theta_{3}\left(f_{\tau },f_{\eta }\right)$ is described in Equation (11).
